# Jeremiah Noah Morris y la epidemiología social

**DOI:** 10.18294/sc.2024.5329

**Published:** 2024-11-26

**Authors:** Hugo Spinelli, Andrés Trotta, Marcio Alazraqui

**Affiliations:** 1 Doctor en Salud Colectiva. Docente-investigador, Instituto de Salud Colectiva, Universidad Nacional de Lanús, Buenos Aires, Argentina. hugospinelli09@gmail.com Universidad Nacional de Lanús Instituto de Salud Colectiva Universidad Nacional de Lanús Buenos Aires Argentina hugospinelli09@gmail.com; 2 Doctor en Salud Colectiva. Director, Doctorado en Salud Colectiva, Instituto de Salud Colectiva, Universidad Nacional de Lanús, Buenos Aires, Argentina. doctortrotta@gmail.com Universidad Nacional de Lanús Instituto de Salud Colectiva Universidad Nacional de Lanús Buenos Aires Argentina doctortrotta@gmail.com; 3 Doctor en Salud Colectiva. Director, Instituto de Salud Colectiva, Universidad Nacional de Lanús, Buenos Aires, Argentina. malazraqui@yahoo.com.ar Universidad Nacional de Lanús Instituto de Salud Colectiva Universidad Nacional de Lanús Buenos Aires Argentina malazraqui@yahoo.com.ar

**Keywords:** Salud Pública, Epidemiología social, Desigualdades Sociales, Actividad Física, Public Health, Social Epidemiology, Health Inequalities, Physical Activity

## Abstract

Este artículo explora la vida y la obra de Jeremiah Noah Morris (1910-2009), un pionero en la epidemiología social. Morris fue un defensor de la interpretación social de la salud y la enfermedad, destacando el impacto de las desigualdades sociales en la morbimortalidad. Su obra Uses of epidemiology impulsó el estudio de enfermedades crónicas desde una perspectiva poblacional. Participó en la elaboración del Black Report en 1980, que evidenció las desigualdades en salud en el Reino Unido, y en el desarrollo del concepto de “ingreso mínimo para una vida sana”. Sus investigaciones relacionaron la actividad física con la prevención de enfermedades coronarias, y defendió la función del médico general en la atención de salud pública. Morris consideró la epidemiología como una ciencia histórica destinada a la resolución de preguntas prácticas, y en ese accionar demostró una actitud abierta ante otros saberes, convocando investigadores que desde diferentes disciplinas le permitían aproximarse a la complejidad de los problemas sociales. Este artículo revisa sus contribuciones y debates claves, abordando la relevancia de sus postulados en el contexto actual y el aparente olvido de su legado en la epidemiología moderna.

## Abstract (en)

This article explores the life and work of Jeremiah Noah Morris (1910-2009), a pioneer in social epidemiology. Morris advocated for a social interpretation of health and disease, emphasizing the impact of social inequalities on morbidity and mortality. His work, Uses of epidemiology, promoted the study of chronic diseases from a population-based perspective. He contributed to the development of the Black report in 1980, which highlighted health inequalities in the United Kingdom, and to the concept of a “minimum income for a healthy life.” His research linked physical activity to the prevention of coronary diseases, and he championed the role of community physicians in public health care. Morris saw epidemiology as a historical science oriented towards solving practical problems, and in doing so he showed an openness to other knowledge, including researchers from different disciplines, which allowed him to approach the complexity of social problems. This article reviews his contributions and key debates, addressing the relevance of his ideas in today’s context and the apparent neglect of his legacy in modern epidemiology.

## INTRODUCCIÓN

…la nueva hegemonía gestada por las fuerzas de derecha exige olvidar la historia, desconocerla y por supuesto, dejar de enseñarla.^1^

Jeremiah Noah Morris (1910-2009) impulsó una interpretación social de las formas de enfermar y morir, enfocado en las desigualdades sociales. Su trabajo se expresó en más de 150 artículos científicos, y su libro *Uses of epidemiology*, del que se realizaron tres ediciones^2^^,^^3^^,^^4^, fue un faro en el campo de la epidemiología a nivel internacional. En 1985, se publicó la primera edición de su libro en español, pero con una modificación en el título que reemplazó la noción de “usos” por “aplicaciones” de la epidemiología^5^.

Morris construyó caminos que transitaron muchos de los que buscaban una mirada social sobre los procesos de salud-enfermedad-atención^2^*.* Su nombre y su obra parecieran haber entrado en el olvido en los ámbitos académicos, a pesar de la relevancia de su trabajo, la actualidad de los graves problemas que identificó, y la vigencia de sus preguntas sobre los problemas sociales y del campo de la salud.

Con el propósito de recuperar su memoria y enfrentar el olvido de su persona y su pensamiento, en este artículo nos proponemos abordar su trayectoria de vida a través de los siguientes puntos: sus orígenes familiares; su infancia; sus estudios de medicina; los primeros problemas de salud-enfermedad que llamaron su atención; su participación en la Segunda Guerra Mundial; sus inicios en el campo de la salud pública y la epidemiología; sus trabajos con prestigiosos cientistas sociales, los principales aportes de *Uses of epidemiology*^2^; el *Black Report*^6^*;* el ingreso mínimo para una vida sana*,* los premios y reconocimientos que recibió, y su militancia política. 

Para el desarrollo de los puntos anteriores nos basamos en diferentes materiales como entrevistas, sus publicaciones y presentaciones, y los reconocimientos *post-mortem* en diferentes revistas de epidemiología, historia y salud pública, como también en diarios ingleses, que resaltaron su trayectoria como investigador y como ciudadano comprometido en enfrentar las desigualdades sociales. 

Morris construyó una mirada social sobre las formas de enfermar y morir en las poblaciones, destacando la identidad social de la epidemiología, que incluyó lo biológico, pero no se limitó a esa dimensión, sino que fue más allá, en la búsqueda de elucidar la jerarquía de la cuestión social de los problemas^7^^,^^8^^,^^9^^,^^10^^,^^11^.

En los inicios del siglo XX se originó en el Reino Unido una mirada social de los problemas de salud y enfermedad que demostró que las desigualdades sociales se expresan de diferentes maneras en la sociedad. Esos problemas fueron abordados desde diferentes disciplinas como la epidemiología, las estadísticas, y las ciencias sociales, por figuras como Richard Titmuss (1907-1973); Archibald Cochrane (1909-1988); Richard Doll (1912-2005); Donald Reid (1914-1977), Peter Armitage (1924-2024); y Peter Brereton Townsend (1928-2000). Esa corriente de medicina social tuvo en Jeremiah Noah Morris a uno de sus principales impulsores^7^^,^^11^.

Todos ellos pertenecieron a un momento histórico marcado por la pobreza, las desigualdades sociales y el desarrollo de la ciencia, e incidieron en esas realidades a través de sus posiciones teóricas, sus abordajes metodológicos, y muchas veces por sus posiciones políticas. Sus interpretaciones desde lo social impactaron en el conocimiento científico, y no estuvieron alejados de polémicas tanto en los ámbitos académicos como políticos. 

Milton Terris se preguntó ¿por qué ese movimiento que a inicios del siglo XX recuperó la vieja idea de la salud como una cuestión social ocurrió en Inglaterra^12^, si en esos años había participado en la Segunda Guerra Mundial y no tenía el dinero que sí tenía EEUU? Terris refiere haber escuchado de John Alexander Hamilton Lee la respuesta a su pregunta, quien señalaba que esa orientación social procedía de una conciencia política compartida:

Mi propia interpretación es que los factores políticos son importantes. La razón de que el movimiento comenzara en Gran Bretaña -y es difícil decir por qué no aconteció en otra parte- fue en gran parte porque en el liderazgo del movimiento británico en favor de la medicina social influyó la ideología laborista y socialista. Major Greenwood fue socio fundador de la Asociación Médica Socialista (AMS) en 1930; Richard Doll era socio activo y el propio Ryle sostenía lazos estrechos con la AMS. Jerry Morris era indudablemente laborista. J. A. H. Lee me dijo en una reunión de la Asociación Internacional de Epidemiología celebrada en Yugoslavia, que los que se dedicaban a la medicina social en Gran Bretaña reunían por lo menos dos de tres condiciones: una, eran laboristas; dos, eran escoceses y, tres, habían hecho algo diferente antes de entrar en la medicina.^12^

La fuerte presencia de la epidemiología social en Inglaterra a partir de las décadas de 1950 y 1960, con referentes provenientes sobre todo del Partido Laborista y la Asociación Médica Socialista, no tuvo influencia relevante en la medicina social, epidemiología social o salud colectiva que se dio en América Latina en las décadas de 1960 y 1970, con bases teóricas relacionadas al marxismo en versiones tanto estructuralistas (influencia francesa de Louis Althusser), como culturales (influencia italiana, de Antonio Gramsci)^13^^,^^14^^,^^15^^,^^16^^,^^17^^,^^18^^,^^19^^,^^20^^,^^21^^,^^22^^,^^23^^,^^24^. 

Fueron múltiples los problemas sociales y de salud que llamaron la atención de Morris, algunos se transformaron en ejes de sus investigaciones, otros tuvieron menos jerarquía en sus antecedentes científicos, pero no en sus preocupaciones. Entre los problemas más importantes se encuentran: las desigualdades sociales y su relación con el tabaquismo, el ejercicio físico, la contaminación ambiental, la enfermedad coronaria, el cáncer de pulmón, la úlcera péptica, la educación médica, la figura del médico general, los servicios sociales, las dietas, y el ingreso mínimo para una vida sana. 

Morris, al final de su vida, se lamentó de no estudiar el problema social de las violencias, muchas de las cuales presenció en su infancia en Glasgow, donde la violencia sobre las mujeres estaba naturalizada^7^, y se mostraba sorprendido que los epidemiólogos no la hayan abordado como un problema de salud pública^25^.

## ALGUNOS DATOS BIOGRÁFICOS

En los primeros años del siglo XX, su padre Nathan (un erudito lingüista hebreo del cual se desconoce el apellido original) y su madre, Ann Yoselofsky, escaparon de los pogromos zaristas del este de Polonia rumbo a Inglaterra. Al arribar, el padre se cambia el apellido por Morris, que era el apellido del capitán del barco en el cual habían realizado el viaje. Con ese cambio, buscaban escapar de la estigmatización que habían sufrido por ser judíos. El matrimonio tuvo tres hijos, Jeremy, apodado Jerry, nacido en Liverpool el 6 de mayo de 1910; Max, nacido el 15 de agosto de 1913; e Isaías, nacido el 19 de junio de 1917^3^^,^^10^. Isaías, se recibió de médico y murió en 1948 en el marco del conflicto árabe-israelí; Max fue uno de los profesores y pensadores de izquierda más influyente de su generación, tuvo fuerte militancia gremial y fue presidente del National Union of Teachers^8^^,^^9^.

La familia se instaló en Glasgow, ciudad con la cual Morris siempre se identificó. Su barrio de la infancia estaba marcado por la pobreza. Allí Morris padeció raquitismo, que le dejó como secuela una lesión en la muñeca que solía mostrar a los estudiantes cuando contaba su origen social^26^.

El raquitismo fue una epidemia en la Gran Bretaña victoriana y se extendió hasta la década de 1930, especialmente entre los pobres de las ciudades industriales. […] La reaparición del raquitismo en Glasgow generó una ansiedad considerable. El raquitismo desapareció de la ciudad en los años de la posguerra, y recién en 1959 el número de casos comenzó a aumentar nuevamente hasta alcanzar en 1963 diez casos y, en 1964, 38 casos en niños nativos, particularmente en las zonas más pobres de Glasgow donde el raquitismo se asociaba al hacinamiento, a las familias numerosas, y a las nubes de humo que impedían la entrada del sol.^4^

A los 16 años ingresó al Partido Laborista (Labour Party), el cual abandonó cuando tenía noventa años por su desacuerdo con la invasión del Reino Unido a Irak, el 20 de marzo de 2003^25^, desde ese momento pasó a votar al Partido Verde (Green Party)^7^.

En 1939, se casó con Galina (Galia) Schuchalter, una exiliada rusa, quien fue su compañera durante más de 50 años y murió el 28 de octubre de 1997, en Londres. Ellos adoptaron dos hijos, David y Julie, cuyos padres habían sido asesinados en los campos de concentración^7^. 

Jeremiah Morris murió por causa de una neumonía, en Londres, a la edad de 99 años, el 28 de octubres de 2009. Su muerte tuvo amplia repercusión en el mundo académico y en los medios de comunicación, publicándose múltiples notas de reconocimiento sobre su figura^7^^,^^8^^,^^9^^,^^11^^,^^26^.

### Su trayectoria

En 1928, la familia se mudó a Londres, donde obtuvo su título de *Bachelor of Arts* en la Glasgow University*.* En 1934, se graduó de médico en el College Medical School de la London’s University y obtuvo su membresía en el Royal College of Physician^11^. Su interés por estudiar epidemiología y salud pública en la London School of Hygiene & Tropical Medicine (LSHTM) tuvo que posponerlo debido a la Segunda Guerra Mundial, y recién en 1947, al terminar la guerra, pudo ingresar y realizar su primera formación en epidemiología^7^^,^^9^. Al año siguiente, al fundarse la unidad de medicina social del Medical Research Council, pasó a ser su director, invitado por Horace Joules, médico y activista de la medicina preventiva^25^^,^^27^. En esa unidad, trabajó con profesores como Richard Titmuss, Bob Logan, Maurice Backett y Austin Heady, con quienes transformaron la institución en un faro internacional de la medicina social^27^. 

A partir de 1956, la sede de esa unidad se trasladó al London Hospital, donde Morris fue nombrado catedrático de medicina social. Entre los años 1967 y 1975 la unidad pasó a la London School of Hygiene & Tropical Medicine, donde Morris fue profesor de salud pública hasta su jubilación^8^. Allí, en 1970, lanzó la Maestría en Medicina Social impartida conjuntamente con profesores de la London School of Economics^28^. Esa maestría estaba destinada a formar la nueva generación de médicos con una mirada social sobre la salud, y reemplazó al obsoleto Diplomado en Salud Pública^29^. En esa maestría se capacitó una nueva generación de líderes con distintas formaciones que iban desde las ciencias sociales hasta la investigación operativa. Algunos de sus graduados fueron Iain Chalmers, June Crown, Beulah Bewley, John Ashton y Michael Marmot todos ellos discípulos de Morris^8^. 

El accionar de Morris fue mucho más allá de una mirada médica, ya que su compromiso social lo llevó a conformar equipos interdisciplinarios para estudiar problemas epidemiológicos relacionados con las desigualdades sociales. Richard Titmuss y Peter Townsend son algunos de los cientistas sociales que compartieron trabajos con él, tal como veremos más adelante en otros apartados. 

Morris fue asesor de los gobiernos laboristas en los años 1960 y 1970^30^. También fue un miembro clave de muchos comités de salud de la posguerra, como el primer comité del Royal College of Physicians a finales de la década de 1950, abocado a temas de salud, tabaquismo y contaminación ambiental, lo que lo llevó a centrarse en la economía del tabaquismo, y en los medios de comunicación, una idea muy novedosa para la época^7^. También participó activamente del Committee Seebohm^31^, sobre servicios sociales personales, y del Committee Hunter^32^, sobre el consumo de tabaco y sus efectos a la salud. Estos comités resultaron de vital importancia en la reestructuración del National Health Service (NHS), en la jerarquización del médico general y en establecer nuevos departamentos de servicios sociales a nivel local. A principios de la década de 1980, Morris fue presidente del National Committee on Nutrition Education, cuyo informe final molestó a la industria de alimentos y motivó un duro enfrentamiento con el gobierno de Margaret Thatcher^8^.

Morris influyó en las trayectorias de profesionales de varios países. En un seminario realizado el 21 de julio de 2000 en la London School of Hygiene & Tropical Medicine, al celebrarse los 90 años de Morris^33^, Michael Marmot reconoció que fue para él como un padre y que le dio mucho más de lo que él pudo darle. También recordó que “Jerry fue la única persona que me dijo que debería ver más televisión, no menos”^27^. Ello evidencia la preocupación de Morris sobre la jerarquía de los medios de comunicación y el rol que estaban teniendo en las temáticas de salud, enfermedad y atención^27^. Todos recordaban a Morris como “un riguroso con la precisión” al momento de entregar un documento o un artículo para ser publicado. Circulaba en los pasillos de la London School of Hygiene & Tropical Medicine la anécdota de que una vez su secretaria llegó a mecanografiar 32 veces uno de sus artículos. Otra singularidad de Morris, fue que nunca condujo un automóvil, y que siempre buscaba quien lo acercara a su hogar, o caminaba largos trayectos en los cuales conversaba con sus discípulos^27^. 

En el año 2008, en una reunión de estudiantes de las promociones de 1973 a 1975, en su casa, Morris refirió que se sentía alentado por los éxitos que sus estudiantes habían logrado en sus vidas, entre ellos se encontraba Iain Chalmers, fundador de la Colaboración Cochrane^7^.

El historial de Morris demuestra que estaba profundamente motivado por mejorar el bienestar humano. Pero para él, no era suficiente presentar las pruebas, sino que había que implementarlas y marcar con ello la diferencia. A esa acción la denominó “activismo en salud pública”^25^. 

A lo largo de su vida, Morris recibió dos medallas Edwin Chadwick, y el premio trianual de la London School of Hygiene & Tropical Medicine, en los años 1947 y 2002, entre otras distinciones^7^^,^^34^. A los 90 años, Morris se sentía feliz de ser miembro fundador de Move4Health, constituido para presionar a favor de fomentar la actividad física^9^. 

Su hijo David, en el discurso que dio en el funeral de su padre, señaló que además de sus caminatas habituales a su oficina, su padre hacía ejercicio todos los días, y en los últimos tiempos, los realizaba en una bicicleta estática. Además, asistía semanalmente al teatro, a la ópera, a conciertos, leía las principales revistas sobre medicina, salud pública y política, y era un gran lector de novelas policíacas^35^^,^^36^.

### La Segunda Guerra Mundial (1939-1945)

En el marco de la Segunda Guerra Mundial, Morris fue incorporado al ejército británico como médico militar. Esa dura experiencia no logró aplacar su inquieto espíritu de investigador, ni su interés por la ciencia, los cuales se mantuvieron muy activos como lo evidencian tres hechos que recuperamos y que transcurrieron en esos años. 

En 1944, ya incorporado al Royal Army Medical Corps con el cargo de mayor, publicó para la Association for Education in Citizenship un texto titulado *Health*^37^, el cual formaba parte de una serie de manuales para grupos preocupados e interesados en discutir los problemas sociales, económicos y políticos de la Segunda Guerra Mundial. El problema central que el texto aborda es la función social de la salud, utilizando estadísticas que evidencian la mala situación de salud en Gran Bretaña. Morris destacó las cuestiones socioeconómicas como responsables del empeoramiento de los indicadores. El texto no se limitaba a la morbimortalidad, sino que también presentaba argumentos a favor de un servicio de salud universal financiado con impuestos y en las conclusiones exhortaba a los lectores a involucrarse en la comprensión de la salud de las comunidades en las que vivían a través de una serie de preguntas, que en su mayoría mantienen total vigencia:

…en tu propia comunidad. ¿Cuántos médicos y dentistas hay? ¿Cómo están distribuidos? ¿Cuántas clínicas, cuántos hospitales? ¿Cómo es ser paciente ambulatorio? ¿Cuántas fábricas tienen personal médico? ¿Cuántos niños están vacunados contra la difteria o la viruela? ¿Cuánta leche se pasteuriza o se somete a la prueba de la tuberculina? ¿Cuánto gasta tu municipio en servicios de salud? ¿Qué ha hecho el municipio con sus atributos como poder ejecutivo? ¿Cuánto humo hay en el aire? ¿Cuánto hacinamiento? ¿Cuántos parques, piscinas y campos de juego hay? ¿Cuánto cuesta y cuánto tiempo toma llegar al área rural para pasar un día de descanso? ¿Cuántas fábricas y comercios dan vacaciones pagas? ¿Cuáles son las tasas locales de mortalidad infantil, tuberculosis en personas jóvenes, difteria, etc.? En todos estos aspectos, ¿cómo se compara su comunidad con los distritos vecinos, con todo el país, con la mejor zona? ¿Por qué existen estas diferencias?^37^

El segundo hecho de este apartado se relaciona con los pacientes que Morris había atendido en el University College Hospital antes de la guerra. Allí había observado, junto a Richard Titmuss, fuertes relaciones entre la situación social de los enfermos y la enfermedad cardíaca reumática. Durante la guerra, a pesar de la distancia que los separaba, Morris y Titmuss continuaron trabajando a través del intercambio postal, y como producto de esas comunicaciones se publicaron tres artículos que fueron aclamados por el pionero de la medicina social, John Ryle, como el primer ejemplo de una medicina social práctica^8^. Los artículos publicados en *The Lancet* trabajaron el reumatismo juvenil^38^, la cardiopatía reumática^39^, y la úlcera péptica^40^, demostrando la relación entre esas enfermedades y la cuestión social. Las causas de esas enfermedades eran, hasta entonces, prácticamente desconocidas^9^.

En los últimos años de la Segunda Guerra Mundial, Morris fue destinado a India por el Royal Army Medical Corps^7^^,^^25^, primero a Bangalore en 1943, y luego a Assam en 1944. De esas experiencias surge un hecho muy singular que realizó con la penicilina, de la cual había leído mucho, pero nunca la había usado. En un momento en el que hay dos soldados con infecciones graves, buscan y consiguen penicilina, de manera no oficial, y la usan en ambos pacientes, y logran salvarles la vida, por lo que consideraba que él había sido el primero en usar penicilina en el campo de batalla^7^^,^^8^^,^^41^.

### Su formación como epidemiólogo y su concepción de la epidemiología

En una entrevista realizada por David L. Smith, del Department of Social Medicine, University of Bristol^25^, Morris explicitó que los epidemiólogos más importantes de su vida fueron Major Greenwood (Reino Unido, 1880-1949), Joseph Goldberger (EEUU, 1874-1929), y Austin Bradford Hill (Reino Unido, 1897-1991). De Major Greenwood rescató la calidad de su escritura, y la capacidad de abordar el problema del cáncer desde una mirada poblacional, lo cual influyó en su trabajo en Nottingham, donde examinó a todos los trabajadores del ayuntamiento. Como no conocía a Greenwood, fue a verlo, y compartieron una larga conversación sobre cómo enfocar esa investigación^25^. Morris aprendió del trabajo de Goldberger y Sydenstriker sobre la pelagra, incluido los aspectos experimentales^42^. Goldberger trabajaba con Edgar Sydenstriker, a quien Morris consideraba con una visión social mayor que Goldberger^25^. 

Edgard Sydenstriker (1881-1936), considerado un pionero de la economía de la salud^43^, realizó estudios de posgrado en la Chicago University y en la Johns Hopkins University. En 1915 se unió al Servicio de Salud Pública de EEUU, donde trabajó con Benjamin S. Warr, investigando la situación económica y de salud de los trabajadores de las fábricas textiles en la ciudad de Nueva York, así como el seguro de enfermedad en Europa. Sydenstricker y Wade Hampton Frost comenzaron en 1918 a investigar la influenza con herramientas estadísticas. Sydenstricker fue nombrado jefe de la Oficina de Investigaciones Estadísticas en 1920 y comenzó la Encuesta de Morbilidad de Hagerstown al año siguiente que más tarde se convirtió en la National Health Survey de EEUU^43^^,^^44^. El otro estadístico que influenció a Morris fue Austin Bradford Hill, quien aportó claridad metodológica de los usos epidemiológicos que se podían hacer con la estadística. Es interesante señalar que Bradford Hill no tenía doctorado, aunque fue director del Department of Medical Statistics and Epidemiology de la London School of Hygiene & Tropical Medicine.

Sobre su decisión de ser epidemiólogo, Morris refiere que cuando era un joven graduado en Medicina, ya se interesaba por la cardiopatía reumática, a la que terminó por adoptar como su problema favorito. Él reconoce la influencia que tuvieron en él los pacientes que había atendido en el University College Hospital, donde había observado muchas cuestiones sociales relacionadas con esa enfermedad. Ese interés lo llevo a analizar las estadísticas del Registrar General’s Department del Reino Unido sobre la mortalidad por enfermedades cardíacas entre los niños y relacionarlos con las condiciones sociales. Esos trabajos los realizó con Richard Titmuss, y representaron para Morris su puerta de entrada, a través de la epidemiología, en la medicina social y la salud pública^25^. 

Él consideraba que la contribución más importante de la epidemiología a la sociedad había sido evidenciar que la salud y la enfermedad son cuestiones tanto sociales como biológicas, destacando la importancia de la mirada poblacional junto con la clínica y el laboratorio, en cualquier discusión seria sobre etiología e historia de una enfermedad. Para Morris esos cambios representaban un nuevo paradigma^25^.

Al ser interrogado sobre los consejos que le daría a un epidemiólogo que comienza su carrera, Morris respondió que debería alcanzar capacidades en estadísticas, medicina, ciencia molecular o sociología, y de todas ellas consideró que la más importante era la sociología, reflexionando que a lo largo de los años, muchas veces, la medicina en sus investigaciones descubrió conceptos sin saber que la sociología los habían descubierto antes^25^. Es indudable que en esa respuesta hay un gran reconocimiento a su amigo Richard Titmuss. En 2004 Morris afirmó en relación con la epidemiología:

Hoy en día, la epidemiología es una ciencia madura, con revistas, libros de texto, títulos y departamentos universitarios. Es común que haya un aspecto epidemiológico dentro de los artículos clínicos. En mi época no había nada parecido. Fuimos creando la epidemiología a medida que avanzábamos.^9^

El pensamiento epidemiológico de Morris no ignoró la clínica, pero si marcó claras diferencias entre la clínica y la epidemiología:

Puede haber cientos o miles de pacientes en los “libros” de una clínica diabética universitaria, pero las cifras por sí solas no garantizan que reflejen la ocurrencia de trastornos renales, retinianos o isquémicos en la diabetes y no simplemente en un grupo particular, y probablemente bastante indefinible, de diabéticos. A menudo se seleccionan pacientes de un área, con casos sospechosos de padecer estas “complicaciones” para remitirlos a clínicas universitarias, etc. Para empezar, sería mejor asegurarse de que se estudia a todos los pacientes con diabetes definible de una población amplia, en consultas generales, por ejemplo; se trata de una cuestión epidemiológica y esa es la forma correcta de plantearla.^2^

## SUS CONTRIBUCIONES

### El ejercicio físico: “la mejor inversión en salud”

Al finalizar la Segunda Guerra Mundial se observaba un aumento sostenido de la mortalidad por infartos de miocardio, sin ninguna explicación científica, ante lo cual muchos caracterizaron la situación como epidémica. En ese contexto, Morris, quien era un fumador regular, conoció el informe de Sir Richard Doll^45^ y dejó de fumar, con cierta dificultad, y empezó a caminar regularmente, práctica que continuó hasta los últimos días de su vida. A medida que fue envejeciendo, él observó que el ejercicio era beneficioso para las personas mayores^7^. Para explicar su interés por el ejercicio, Morris remitía a una vivencia con su padre, quien los llevaba a él y a sus hermanos a dar largos paseos, recompensándolos al final con un helado para cada uno^7^.

Antes de la década de 1950, el ejercicio físico se consideraba necesario para lograr y mantener la aptitud para el trabajo, la acción militar o el rendimiento deportivo. Pero los trabajos de Morris demostraron que la actividad física además era muy importante para mantener la salud^9^. Morris señaló que, en Occidente, éramos la primera generación, en la historia de la humanidad, en la que la población tenía que hacer ejercicio para estar saludable^46^. Morris fue pionero en la década de 1960 en realizar largas caminatas, decía que en Hampstead Heath, cerca de su casa, “la gente pensaba que yo era un loco”^7^. Morris estaba muy adelantado a su tiempo, como puede apreciarse en el mensaje de su artículo de 1949, en el que sostiene que “el ejercicio era la mejor inversión en salud pública que se podía realizar”^47^. Las dimensiones placenteras y terapéuticas de caminar, tanto en lo psicológico como en lo social, si bien son evidentes, han sido sorprendentemente poco estudiadas, señaló Morris en 1997^7^. Tuvieron que pasar muchos años para que el impacto del ejercicio físico comenzara a sentirse en los hábitos y la vida diaria de las personas en el mundo desarrollado.

Morris fue un decidido investigador de los problemas originados por el aumento del número de infartos de miocardio, y para ello organizó un amplio estudio para examinar personas de diferentes ocupaciones. En dichos trabajos encontró que los conductores sedentarios de los autobuses de dos pisos de Londres tenían tasas más altas de enfermedades cardiovasculares que los inspectores que subían las escaleras^48^. También que los carteros que entregaban el correo en bicicleta, o a pie, tenían menos ataques cardíacos que los hombres sedentarios que trabajaban detrás de mostradores como los telefonistas y los administrativos^49^. Esto señalaba que la falta de actividad física representaba un riesgo significativo para la enfermedad coronaria. En 1958, publicó un trabajo en el que mostraba que los movimientos lentos como los de la jardinería ayudaban muy poco en la prevención de la enfermedad coronaria, y que el ejercicio debía ser más vigoroso para ser útil^50^. 

Morris utilizó medidas simples en sus investigaciones; por ejemplo, en el trabajo con los conductores de autobuses, tomó el tamaño de la cintura de los pantalones para demostrar la relación principal entre el ejercicio y las enfermedades cardíacas, en lugar del tamaño relativo de la cintura. Esa investigación duró tres años y al momento de ser publicado no faltaron las miradas incrédulas que cuestionaron los datos^49^^,^^51^^,^^52^. Morris consideró años después que, al momento de ser publicado, ese artículo no tuvo ningún impacto en la cardiología inglesa, que no tenía idea de cómo manejar el tema. Morris consideraba que esas críticas lo dejaron en ridículo, aunque reconocía que sí tuvo impacto en los medios de comunicación^25^. Morris pasó muchos años realizando diferentes estudios, antes de atreverse a publicarlos, y cuando los publicó los presentó como hipótesis^25^. 

Durante las décadas siguientes, impulsó investigaciones para relacionar la actividad física y la salud, como la realizada con funcionarios públicos en 1970^53^^,^^54^. Sus trabajos proporcionaron un estímulo para que Paffenbarger y colaboradores realizaran un estudio en las décadas de 1970 y 1980 sobre exalumnos de Harvard University^55^^,^^56^^,^^57^^,^^58^^,^^59^, y también un seguimiento longitudinal sobre la aptitud física en el Aerobics Center en Dallas, desde finales de la década de 1980^9^.

Fuera de la epidemiología, el primer indicio de que estaba surgiendo un nuevo campo relacionado con la salud pública en el Reino Unido provino del English Sports Council, que comenzó a promover el deporte y las actividades cotidianas en beneficio de la salud^9^. Por su parte, Bassey y Fentem realizaron la primera evaluación integral de la influencia del ejercicio en la salud de las personas, en la que destacaban los estudios de Morris^60^. No mucho tiempo después se impulsó que los profesores de educación física se dieran cuenta de la importancia de la actividad física para la salud en la niñez y comenzaran a aplicarla en los planes de estudios. Sin embargo, la ciencia del ejercicio tardó en desarrollarse y conservó el énfasis en las ciencias del deporte, centrándose en la fisiología del entrenamiento, la biomecánica del movimiento y la psicología del deporte y su rendimiento. No fue hasta 1993 que la British Association of Sport Sciences añadió “*Exercise*” a su título y conformó la sigla BASE. Y recién en el año 2000, la British Association of Sports Medicine añadió la palabra medicina a la sigla que lo identificaba, y pasó a ser BASEM^9^. 

En 1988, le solicitaron a Morris que presidiera la Activity and Health Research Board, una entidad benéfica destinada a realizar y promover investigaciones sobre el ejercicio, el *fitness* y la salud. La primera tarea fue realizar la English National Fitness Survey, un estudio a gran escala sobre la actividad física y el *fitness* en una muestra aleatoria de adultos en Inglaterra en 1990^61^. Los resultados revelaron que aproximadamente el 70% de los adultos no eran activos y ello afectaba su salud. Lo más sorprendente fueron los niveles informados de aptitud para caminar, el 30% de los hombres y el 49% de las mujeres de 55 años o más superaron el 70% de su frecuencia cardíaca máxima estimada al caminar a 4,7 km/h en llano. Las proporciones aumentaron al 70% y al 91% para hombres y mujeres, respectivamente, al caminar a 4,7 km/h en una pendiente del 5%. Los resultados revelaron que una gran proporción de adultos de mediana edad y mayores ni siquiera estaban en forma para realizar las actividades de la vida diaria^9^. Los resultados de la encuesta dieron lugar a una serie de acontecimientos importantes a nivel nacional, instalando el tema en la agenda pública^9^.

En 1991, se llevó a cabo la primera encuesta de salud anual en Inglaterra sobre diversos aspectos de la salud del país, en la que se preguntaba a las personas sobre su actividad física, utilizando un instrumento similar al desarrollado por Morris para la English National Fitness Survey^27^. En 1996, se lanzó una campaña en los medios de comunicación para educar a la población sobre los beneficios de la actividad física para la salud^28^. 

En los Juegos Olímpicos de 1996, Morris y Ralph Paffenbarger fueron honrados con una medalla de oro olímpica en reconocimiento a la excelencia en la ciencia del deporte y el ejercicio, y a sus estudios sobre cómo el ejercicio reduce las enfermedades cardíacas. 

El 11 de septiembre de 2009, Simon Kuper publicó en el *Financial Times* un artículo sobre el trabajo de Morris que tituló *The man who invented exercise*^35^*.*

### Richard Titmuss: desigualdades sociales y enfermedades

En 1938, Titmuss publicó *Poverty and population*^62^ que se centró en las diferencias regionales entre el norte y el sur del país. Fue a través de la lectura de ese libro, que Morris conoció a Titmuss, identificando intereses y principios muy similares a los suyos. Esto dio inicio a lo que Ann Oakley llamó “una asociación de trabajo inusualmente vital”, tanto en investigación como en acción política, que duró hasta su muerte^7^. 

Titmuss se crió en el campo y abandonó la escuela preparatoria a los 14 años de edad sin calificaciones formales, debido a una enfermedad que limitó su asistencia escolar. Existen sobre esa etapa de su vida relatos que constituyen lo que se conoce como el “mito” de Titmuss, el cual fue construido por Margaret Gowing, antigua compañera de trabajo de Titmuss. Margaret tuvo como fuente de sus dichos a Kathleen Caston Miller, esposa de Titmuss, quien tenía una mala relación con su suegra, que es quien le transmite a Gowing que la madre de Titmuss era “incompetente en el ámbito doméstico”, y que el padre había fracasado como granjero. Esta visión es la que Stewart^63^^)^ recoge en su libro, y es la misma que figura en *The Oxford Dictionary of National Biography*^64^. Esos relatos son diferentes a las conclusiones que obtiene Ana Oakley, hija del matrimonio de Titmuss y Caston Miller, al reconstruir la vida de sus abuelos, donde narra cuestiones muy diferentes, y que contradicen el mito de Titmuss^65^.

Titmuss fue un autodidacta, trabajó durante 16 años como actuario para una gran compañía de seguros, al mismo tiempo que se interesaba por temas sociales a través de lecturas y debates, que volcaba en sus escritos. Sus preocupaciones iniciales se relacionaban con cuestiones como los seguros y la estructura de edad de la población, la migración, el desempleo, el rearme, la política exterior y el movimiento por la paz. 

Morris y Titmuss trabajaron los problemas de salud como hechos sociales y pensaron formas de atención basadas en concepciones marcadas por el espíritu de la posguerra, la creación del National Health Service (NHS), entre otras iniciativas como la medicina preventiva, la epidemiología social, y lo que más tarde se llamaría la sociología de la salud y la enfermedad. Sus preocupaciones se centraron en cuestiones de justicia social^7^.

En 1939, Titmuss publicó en colaboración ***Our food problem: A study of national security*** y, en 1950, *Problems of social policy*. Esas y otras publicaciones fortalecieron su reputación, ganando una cátedra en la Escuela de Economía de Londres, apoyado por el sociólogo Thomas Humphrey Marshall. Su último libro, *The gift relationship*, expresó su propia filosofía del altruismo en la política social y las políticas de salud, y enfatizó su preferencia por los valores del servicio público sobre las formas privadas o comerciales de atención^66^. Años más tarde, fundó la disciplina académica de Administración Social, conocida actualmente como Política Social, y ocupó esa cátedra en la London School of Economics.

Sus libros y artículos de la década de 1950 ayudaron a definir las características del Estado de bienestar de Gran Bretaña después de la Segunda Guerra Mundial, en concordancia con las contribuciones de Gunnar Myrdal en Suecia^7^.

La relación entre Morris, tanto con Titmuss, como con Brian Abel-Smith, ambos de la London School of Economics, tuvo un papel clave en el desarrollo de las políticas sociales y de salud^8^, sobre todo, bajo los gobiernos laboristas del Reino Unido de la década de 1960, y anticiparon los servicios de salud ligados a los territorios y los enfoques de investigación centrados en políticas^7^.

La relación entre las enfermedades y la cuestión social llevó a que Morris y Titmuss pensaran un libro sobre medicina social, del cual solo escribieron algunas notas^7^. El trabajo conjunto entre ambos produjo un rico intercambio desde el que impulsaron diferentes asuntos como la participación de Morris en el Seebohm Committee sobre el futuro de los servicios sociales personales y la participación de Titmuss en el Todd Committee, sobre educación médica en 1968^8^. Titmuss era un gran fumador y murió de cáncer de pulmón el 6 de abril de 1973.

### Los usos de la epidemiología

En 1955, en el *British Medical Journal,* Morris, anticipó los contenidos del libro que saldría al año siguiente, y que se transformaría en un modelo político-técnico para las actividades de salud pública^67^. *Uses of epidemiology*^2^ significó un hito para la epidemiología y el estudio de las desigualdades sociales en salud. Fue el primer libro de epidemiología no abocado a las enfermedades infecciosas^30^. El libro estuvo dedicada a su compañera Galia, y tuvo otras dos ediciones, en los años 1964 y 1975, que ampliaron sus contenidos^3^^,^^4^.

En la presentación del libro, Morris caracterizó a la epidemiología como una forma:

“...de hacer preguntas y obtener respuestas que planteen más preguntas: es decir, como una forma de aprender”.^9^

*Uses of epidemiology*^2^ tuvo gran influencia en la salud pública de la época y en el desarrollo de las estrategias de prevención para el control de enfermedades no transmisibles. La epidemiología de las enfermedades crónicas y el estudio de la distribución de las enfermedades en las poblaciones se transformaron en la base de una nueva perspectiva de salud pública. 

El libro tiene un fuerte soporte en información estadística presentada, en general, como series temporales que abarcan varias décadas, lo cual le otorga una fuerte relevancia a las conclusiones y observaciones obtenidas, enmarcándolas en procesos histórico-culturales. Las tablas y gráficos ocupan casi la mitad de las páginas del libro.

Una premisa central del libro es que en epidemiología se estudian poblaciones y no simplemente individuos o casos particulares del grupo. Y el estudio de las poblaciones exige un diagnóstico dinámico que acompañe los cambios sociales y los problemas que surgen en esos cambios y que se suman a los viejos problemas no resueltos^2^. Y en función de esos diagnósticos se pregunta:

¿Cuáles son las implicaciones para la salud pública de que haya más mujeres casadas saliendo a trabajar?, ¿y menos hombres mayores? ¿De la creciente urbanización y suburbanización? ¿Del rápido crecimiento de nuevas ciudades? ¿De las zonas sin humo (todavía con azufre)? ¿De la construcción de nuevas centrales eléctricas? ¿De la menor actividad física en el trabajo y de la mayor pereza corporal en general? ¿De las aceleradas transformaciones en la industria? ¿De la perspectiva de una era del ocio? ¿O del crecimiento de los medios de comunicación y del uso que se hace de ellos? ¿Del examen “*eleven-plus*”? ¿De los más de 1.000 vehículos motorizados que se suman por día? ¿Del aumento del consumo de azúcar; nuestro asombroso gusto por lo dulce (comemos más per cápita que cualquier otra población)? ¿Del abaratamiento de las comidas con grasas? ¿De la multiplicación de interferencias con los alimentos? ¿De las numerosas exposiciones físicas y químicas, conocidas y potencialmente peligrosas? ¿Del aumento del tabaquismo en las mujeres? ¿Del prodigioso aumento de los rayos X y los antibióticos?^67^

En el capítulo titulado “Recapitulación”, Morris describe “los siete usos de la epidemiología” que se mantuvieron a lo largo de las tres ediciones del libro^2^^,^^3^^,^^4^, con un solo cambio de posición entre el quinto y el sexto uso, en la tercera edición^26^. Entre los usos que Morris le asigna a la epidemiología aparecen temas extraños para lo que, por entonces, se entendía en la disciplina:

1. En el estudio histórico de la salud de la comunidad y el aumento y disminución de las enfermedades en la población; también potencialidad para hacer “proyecciones” útiles hacia el futuro.
2. Para el diagnóstico comunitario de la presencia, naturaleza y distribución de la salud y la enfermedad entre la población y las dimensiones de estas en incidencia, prevalencia y mortalidad, teniendo en cuenta que la sociedad está cambiando y los problemas de salud son cambiantes.
3. Estudiar el funcionamiento de los servicios de salud. Esto comienza con la determinación de las necesidades y los recursos, sigue con el análisis de los servicios en acto y, por último, intenta hacer una evaluación. Estos estudios pueden ser comparativos entre diversas poblaciones.
4. Estimar, a partir de la experiencia común, las probabilidades y los riesgos de enfermedad del individuo.
5. Contribuir a completar el cuadro clínico, incluyendo todos los tipos de casos en proporción; relacionando la enfermedad clínica con la subclínica; observando los cambios seculares en el carácter de la enfermedad, y su cuadro en otros países.
6. En la identificación de síndromes a partir de la distribución de fenómenos clínicos entre sectores de la población.
7. En la búsqueda de las causas de la salud y la enfermedad, partiendo del descubrimiento de grupos con tasas altas y bajas, estudiando estas diferencias en relación con las diferencias en los modos de vida; y, cuando sea posible, poniendo a prueba estas nociones en la práctica real entre poblaciones.^2^

Los siete usos de la epidemiología incluyeron conceptos que ampliaron el campo de la epidemiología, y la colocaron en diálogo con la cuestión social. De todos los usos señalados por Morris, el menos desarrollado y el más resistido para llevarlo a la práctica fue el tres, que propuso el uso de la epidemiología en los servicios de salud para transparentar los gastos y evaluar la calidad de atención.

En 1952, en una conferencia en la que se abordaban los requisitos de la investigación para la salud y la atención médica coincidieron Morris y Kerr White. Allí, White señaló que relacionar la epidemiología con la atención médica constituía un hito en la historia de la investigación en el campo de la salud. Con el tiempo, esos estudios comenzaron a conocerse como estudios de epidemiología de los servicios y sistemas de salud^68^^,^^69^. En 1959, White solicitó con éxito una beca del Commonwealth Fund, y pasó un año en la London School of Hygiene & Tropical Medicine, donde realizó cursos de epidemiología y estadística con Morris, Bradford Hill -que era el decano-, Donald Reid, y otros profesores^70^. 

La idea de evaluar los servicios de salud estaba en el texto que había escrito Morris durante la Segunda Guerra Mundial^37^, momento en el que también se desarrolló la “investigación operativa”, que contemplaba la evaluación de las acciones clínicas y terapéuticas y de los servicios de salud^71^. 

Morris consideró que investigar las etiologías de las enfermedades era una actividad central de las contribuciones futuras de la epidemiología. Su preocupación estaba en las tasas de mortalidad masculina, ya que no se observaban mejoras entre las décadas de 1930 y 1950; mientras que, para el mismo periodo, las tasas de mortalidad femenina disminuyeron constantemente. Ante ello, se preguntaba ¿cuáles eran los procesos sociales que originaban los cambios en las tasas de mortalidad? Morris consideraba que indicaban la dependencia ambiental de la carga de morbilidad, de allí que incorporara la noción de ecología en sus análisis e interpretaciones. Morris estaba interesado en analizar cómo lo social se volvía literalmente biológico, y consideraba que esa debería ser la tarea central de la epidemiología^9^. 

En *Uses of epidemiology*^2^, de manera precursora, se encuentran diferentes debates que se mantienen vigentes, como los relacionados con los enfoques de riesgo aplicados a nivel individual o poblacional para la prevención de enfermedades, enunciado años más tarde por Geoffrey Rose^72^; la necesidad de realizar ensayos aleatorios a gran escala para evaluar la prevención primaria de las enfermedades; y la inclusión del pensamiento ecológico en la epidemiología^73^^,^^74^^,^^75^^,^^76^. Para Nancy Krieger, Morris presagió en cierta manera la epidemiología del curso de vida cuando señaló que las nociones de hipertensión, aterosclerosis y enfermedad coronaria eran también “problemas pediátricos”, y esto representó avances esperanzadores^4^^,^^9^.

La lectura de su libro *Uses of epidemiology*^2^ pone en evidencia que lo social es inseparable de los modos y estilos de vida:

Todo el modo y el estilo de vida están evidentemente implicados en la clase social: los ingresos y la riqueza, la seguridad financiera; el entorno físico; el tamaño de la familia y el espaciamiento de los nacimientos; cómo se cría a los niños; los niveles medios de cociente intelectual, las oportunidades educativas, el interés y ambiciones de los padres por los hijos; los códigos lingüísticos y los modos de hablar; la imagen de uno mismo, los horizontes y los objetivos a largo plazo; las expectativas de salud y las actitudes ante la enfermedad, la utilización de los servicios. No es de extrañar que, a pesar de ser un compendio que se va marchitando a medida que se elimina la pobreza de subsistencia, se mejoran los puestos de trabajo, se sindicalizan las profesiones, los estatus de deferencia declinan y convergen algunos patrones de consumo -la televisión, la propiedad de la vivienda, las tiendas *Marks & Spencer*, las revistas femeninas, el automóvil, las vacaciones en el extranjero, la juventud y la cultura-, esta distinción por “clase social” siga siendo una poderosa herramienta para explorar los niveles de salud de que disfrutan las personas, los problemas que sufren y cómo los afrontan.^4^

Michael Marmot leyó *Uses of epidemiology*cuando era estudiante de posgrado en la sede en Berkeley de la University of California, y le llamó la atención la palabra “usos” en el título, luego se dio cuenta de que Morris veía la epidemiología como una herramienta de fuerte contenido heurístico que ayudaría a crear una mejor salud^9^. 

Para Nancy Krieger^73^, Morris transformó la epidemiología en una ciencia de la población, pragmática y conceptual, basándose en su concepción de que la mejor manera en que la epidemiología interviene en las investigaciones es mediante mejores métodos, solo que el tipo de métodos en los que Morris pensaba, destaca Krieger, no eran los métodos técnicos de la “epidemiología moderna”. Él pensaba en articular un enfoque metódico para el pensamiento epidemiológico, considerando a la epidemiología como una ciencia histórica, demográfica, pragmática y contextual^73^. Esos conceptos habilitaban su pregunta ¿cuáles son los cambios sociales que subyacen a los cambios biológicos en los patrones observados de mortalidad? Para Krieger esa pregunta está ausente en el pensamiento dominante de la *epidemiología moderna*^73^ y, en ese sentido, señala un divorcio entre las preguntas que la epidemiología hegemónica aborda en sus estudios y las realidades en las que las sociedades viven^67^^,^^73^, planteando con ello dos visiones epidemiológicas diferentes: una contextualizada, en la que incluye a Sydenstricker^44^ y Morris^2^, y otra descontextualizada en la que ubica a MacMahon *et al*.^77^ y Rothman^78^.

Para Naomar Almeida, Morris propuso “nociones abstractas como salud y enfermedad para la ciencia epidemiológica”, otorgando un privilegio a lo colectivo (lo poblacional). Almeida, al igual que Krieger, encuentra que las propuestas de Morris fueron sustituidas por nociones idealistas y abordajes instrumentales, coincidiendo con Krieger en la crítica a los tradicionales libros de epidemiología que se usan como referencias, tanto en la enseñanza como en la investigación, y que son hegemónicos en la epidemiología^77^^,^^78^^,^^79^^,^^80^. Esas publicaciones tienden a reducir el objeto de la epidemiología a la prevalencia de enfermedades en la persona, renunciando a cualquier referencia poblacional, fortaleciendo así la idea de que la epidemiología debe usarse para abordajes individualistas, concepción que es funcional a la propuesta de la epidemiología clínica y la medicina basada en evidencias^81^^,^^82^. 

Mientras Morris impulsó el concepto de “usos”, otros epidemiólogos cometieron abusos de las técnicas siguiendo la lógica de la razón instrumental^83^^,^^84^. Reducir la epidemiología a técnicas es negar el pensamiento epidemiológico que se puede encontrar incluso antes de Morris, en John Snow (1813-1858), Joseph Golberger (1874-1929), William Guy (1810-1885), August Hirsch (1817-1894), Wade Hampton Frost (1880-1938) o Edgar Sydenstricker (1880-1936). 

### La estrategia del médico general

Morris fue miembro del Seebohm Committee, sobre servicios sociales personales, y del Hunter Committee, sobre administración médica. Estos comités fueron de importancia central en la reestructuración del servicio de salud y en la designación del médico general, estableciendo nuevos departamentos de servicios sociales en los niveles locales^10^. 

**Figura 1:**
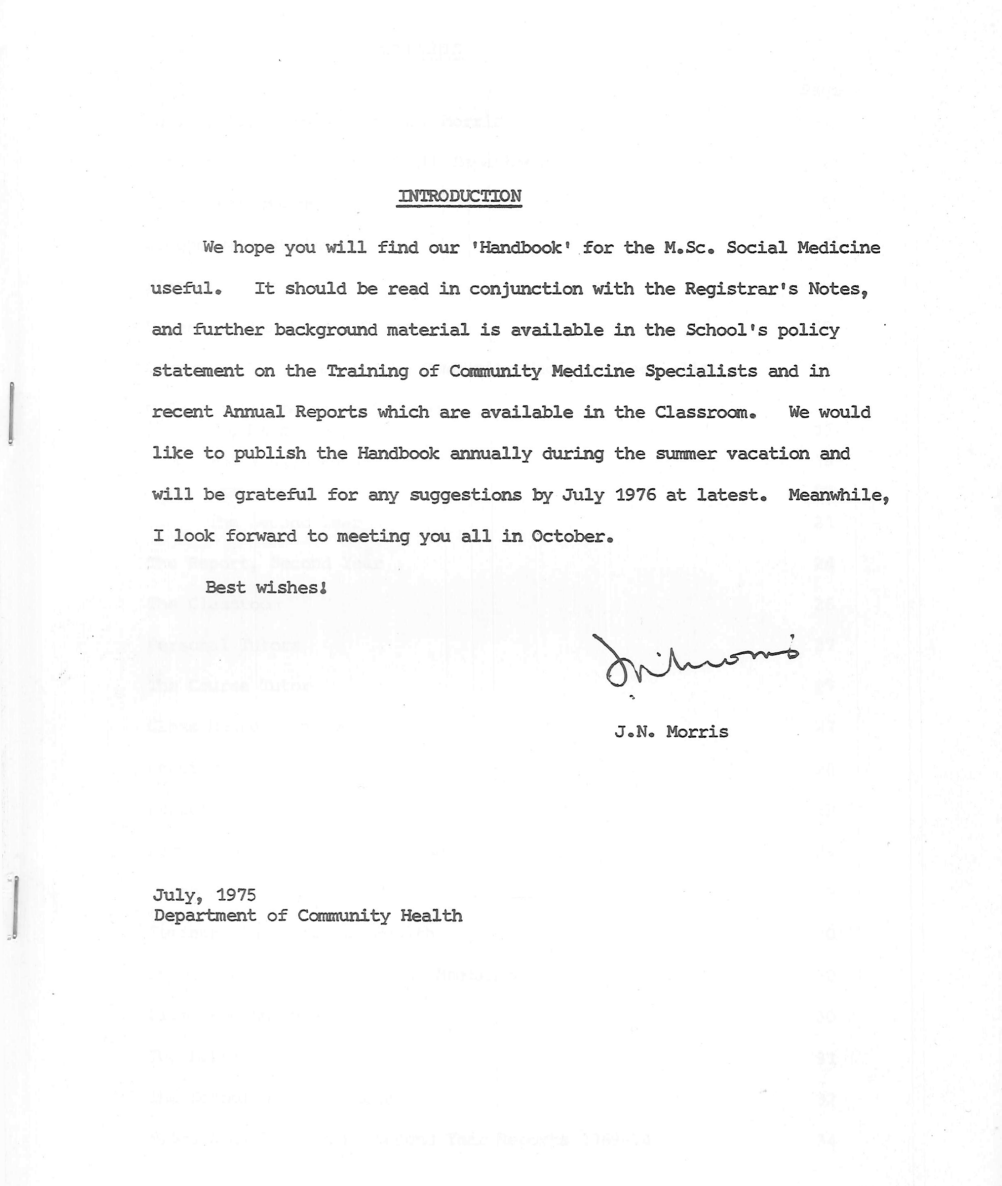
Manual para la Maestría en Medicina Social de la London School of Higiene and Tropical Medicine, 1975.

La visión de Morris sobre el cuidado y la atención de las personas era que debían estar centradas en los “médicos generales” (Figura 1), a los que pensaba como una nueva generación de médicos de salud pública^8^. Para él, el antiguo funcionario médico de salud, que dirigía los servicios de salud dentro del gobierno local debía ser convertido en el nuevo médico general, como un estratega clave en el National Health Service (NHS), responsable del “diagnóstico comunitario”, y que complementaría la medicina comunitaria con la clínica^8^. 

Fuente: Documento donado en 2013 por Mario Testa al Centro de Documentación Pensar en Salud, que puede consultarse en el Fondo Mario Testa. Nota: En la portada mecanografiada se lee “Introducción: Esperamos que encuentren útil nuestro ‘Manual’ para la Maestría en Medicina Social. Debe leerse en conjunto con las Notas del Secretario Académico, y se dispone de material adicional en la declaración de políticas de la Escuela sobre la Formación de Especialistas en Medicina Comunitaria, así como en los Informes Anuales recientes que están disponibles en el Aula. Nos gustaría publicar el Manual anualmente durante las vacaciones de verano y agradeceremos cualquier sugerencia a más tardar en julio de 1976. Mientras tanto, espero encontrarme con todos ustedes en octubre. Saludos cordiales, J.N. Morris, Julio de 1975, Departamento de Salud Comunitaria.

En una conferencia en Johns Hopkins University, Morris definió al médico general como un “administrador de servicios locales, epidemiólogo y consejero comunitario”, además señaló la necesidad de sistemas de información, a los que llamó “servicio de inteligencia eficaz”. Morris pretendía otorgar mayor poder al gobierno local dentro del NHS, lo que en cierta manera se logra en 1973^27^^,^^30^, cuando el médico general se convirtió en el eje ostensible del NHS. Si bien esto buscaba darle mayor racionalidad al uso y funcionamiento de los servicios médicos locales^29^, la realidad no se correspondió con las esperanzas, debido a la pérdida de las raíces de la salud pública vinculada a las comunidades, producto de la medicalización de la sociedad. Así la idea del médico general fue perdiendo la centralidad que proponía Morris^85^.

### Peter Townsend: de la pobreza como privación a las desigualdades sociales

En una entrevista, Townsend reflexionó sobre los inicios de su interés por el estudio de las desigualdades sociales. Su primer recuerdo lo ubica en la década de 1970, pero inmediatamente se rectifica y recuerda que, en realidad, el interés surgió mucho antes, y se debió a Jerry Morris, quien lo invitó a dar una serie de conferencias al London Hospital a inicio de la década de 1960, las cuales dictó por varios años^86^^,^^87^^,^^88^^,^^89^. Para él, las personas importantes en las décadas de 1950 y 1960 fueron Tony Lynes, Jeremy y Sylvia Tunstall, Michael Meacher, Adrian Sinfield, y Jerry Morris^86^. En esa entrevista, Townsend considera a Morris como:

…un hombre voluble e innovador que realizó un trabajo extraordinariamente importante en medicina social. Creo que ha sido una de las figuras claves de la historia reciente británica en medicina social. En parte, lo que ha sucedido es que ha realizado un trabajo muy especializado sobre grupos o enfermedades particulares, que ha logrado ampliar hasta convertirlo en una tesis más generalizada, aunque no creo que él mismo sienta que ha expresado esa tesis en la forma satisfactoria que le hubiera gustado. Publicó un libro de texto sobre epidemiología que ha sido, o lo fue, durante mucho tiempo, el principal instrumento de enseñanza en ese campo […] y ayudó a llamar la atención de la gente sobre algunos de los factores que realmente intervienen en las enfermedades cardíacas.^86^

Peter Townsend (1928-2009) fue un sociólogo británico, profesor de Política Social Internacional en la London School of Economics. Su tema de investigación fue la economía de la pobreza, entendiendo a la pobreza como privación^90^. Su objeto de preocupación, tanto fuera como dentro de la academia, fue la vida de los miembros más pobres y discapacitados de la sociedad. 

Durante la década de 1960, Townsend se desempeñó como miembro del comité de investigaciones del Council for Training in Social Work’s, fue cofundador del Child Poverty Action Group y, en 1965, fue su presidente durante 20 años, además de presidente vitalicio desde 1989. También cofundó Disability Alliance en respuesta al escándalo de la talidomida y la presidió durante 25 años.

### El *Black Report*

El trabajo de Morris sobre las desigualdades sociales fue central en su vida académica. Lo que con el tiempo se conoció como *Black Report* fue encargado en marzo de 1977 por David Ennals, Secretario de Estado de Sanidad y Servicios Sociales de Inglaterra, tras la publicación de un artículo de Richard Wilkinson^91^. Morris no solo estuvo allí, sino que impulsó su creación. En 1980, el gobierno británico lo creó presidido por Sir Douglas Black. Este informe abordó la discusión sobre las desigualdades en la agenda política del Reino Unido. La incorporación del concepto de desigualdades permitió ampliar la discusión a dimensiones que no se contemplaban en el concepto de pobreza, ganando con ello riqueza y complejidad tanto en el análisis como en la discusión del problema.

Morris comenzó a trabajar en el Black Committee en el año 1978^91^*,* y en 1980 el Department of Health and Social Security del Reino Unido publicó el informe “*Inequalities in health*”, conocido popularmente como *Black Report*^6^, por el nombre del presidente del comité, Douglas Black, quien era presidente del Royal College of Physicians*.* El equipo de trabajo estaba conformado por cuatro personas: Sir Douglas Black, Jerry Morris, Cyril Smith y Peter Townsend.

En el marco del Black Committee, a finales de la década de 1970, Morris y Townsend debatieron sobre si las iniciativas sanitarias debían basarse en los servicios de salud o en la comunidad^27^^,^^86^.

Townsend relata que, en 1979, al leer el primer borrador del informe de 50 páginas:

…no me gustó nada, me pareció […] una versión bastante débil de lo que considerábamos un ejercicio muy interesante e importante. Jerry Morris, a quien llamé, estuvo de acuerdo conmigo, y entonces me di cuenta y le dije que dos de cuatro constituían una mayoría porque, en cierto sentido, si dos de los cuatro se oponían, los demás no podían seguir adelante con eso. Así que comuniqué este punto a Sir Douglas y creo que, a regañadientes, aceptó […], y decidimos reescribir el informe y lo tomamos en nuestras propias manos. Stuart Bloom, el secretario del Comité, trabajó muy felizmente con nosotros. Creo que la situación era que él era una persona extraordinariamente versátil y altamente informada que, realmente, estaba haciendo todo lo posible para producir un trabajo de buena reputación […] Y se adaptó muy fácilmente a la idea de que los cuatro autores estarían a cargo de la reescritura.^92^

El informe final de 400 páginas fue escrito en seis meses y se dio a conocer en agosto de 1980. En Inglaterra, se habían realizado elecciones anticipadas y el 4 de agosto de 1979 asumió como primera ministra Margaret Thatcher, con esos cambios en el gobierno, Patrick Jenkin pasó a ser el nuevo secretario de Estado. Luego de unos meses de haberse finalizado, el informe se publicó junto a un comunicado de prensa realizado el viernes anterior al feriado bancario de agosto como una manera de boicotearlo, y se repartieron unas 250 copias en todo el país. El grupo de trabajo del Black Committee se indignó por el procedimiento, y recibió el apoyo de la British Medical Association, de los representantes de la salud pública, de los sindicatos, de *The Lancet* y diversos periódicos, y del sector médico, quienes repudiaron el tratamiento y la burla que recibió el *Black Report*. Luego de un buen tiempo, el *Black Report* fue publicado por la editorial Penguin^93^^)^ conjuntamente con el estudio de Margaret Whitehead, financiado por el Health Education Council *del Reino Unido*, que buscó actualizar aquellos hallazgos*.*

En el prólogo del *Black Report*, el ministro conservador del gobierno de Margaret Thatcher, Patrick Jenkin, señaló como poco realista la opinión que contenía el *Black Report*:

…el Grupo ha llegado a la conclusión de que las causas de las desigualdades sanitarias están tan arraigadas que solo un programa de gasto público importante y de gran alcance es capaz de alterar el patrón. Debo dejar claro que un gasto adicional de la magnitud que podría derivarse de las recomendaciones del informe -la cantidad en cuestión podría ascender a más de 2 mil millones de libras al año- es bastante poco realista en las circunstancias económicas actuales o previsibles, al margen de cualquier juicio que pueda formarse sobre la eficacia de dicho gasto a la hora de abordar los problemas identificados. Por lo tanto, no puedo respaldar las recomendaciones del Grupo. Someto el informe a debate, pero sin que el Gobierno se comprometa con sus propuestas.^6^

En función de lo anterior, y acorde con la visión liberal del gobierno de Thatcher, tomaron la decisión de modificar el término “desigualdades sociales en salud” por el de “variaciones sociales en salud”, estableciendo que una diferencia en la salud no tenía por qué ser injusta. Desde el propio gobierno se decidió restarle importancia al tema y no se realizó ninguna rueda de prensa oficial. A pesar de ello, el informe tuvo un gran impacto en el pensamiento político del Reino Unido, como en otros países que emprendieron estudios similares.

El informe mostró, con gran detalle, hasta qué punto la enfermedad y la muerte se distribuían de manera desigual en la población de Gran Bretaña y sugirió que estas desigualdades se habían ido ampliando en lugar de disminuir desde el establecimiento del National Health Service (NHS), en 1948. Esas importantes desigualdades se expresaban, por ejemplo, en la tasa de mortalidad de los hombres adultos trabajadores no calificados, y que era casi el doble que la de los hombres adultos de la clase social alta. En las tasas de mortalidad por enfermedades específicas, la brecha era aún mayor, por ejemplo, para la tuberculosis, en la que la tasa de mortalidad en la clase social baja era diez veces mayor que la de la clase social alta; para la bronquitis cinco veces más alta y para el cáncer de pulmón y el cáncer de estómago tres veces más alta. Y la tasa de mortalidad neonatal era el doble, y en el primer año de vida cinco veces más alta^6^. 

El informe concluyó que estas desigualdades no eran atribuibles a fallas en el NHS*,* sino más bien a muchas otras desigualdades sociales que influyen en la salud como el nivel de ingresos, el desempleo, la contaminación ambiental, la dieta, las condiciones de trabajo, la educación, la vivienda, el transporte, o el estilo de vida^6^, señalando con ello la dimensión social de los problemas de salud-enfermedad^6^. El informe recomendó una amplia estrategia de medidas de política social para combatir las desigualdades en salud. Por todo ello, el *Black Report* hizo hincapié en la necesidad de abordar las determinantes sociales y económicos que intervienen en las desigualdades en salud, así como el compromiso para implementar políticas gubernamentales transversales para abordarlas. 

Los objetivos del *Black Report* se abandonaron en todo el Reino Unido en 2011, y el efecto de la política en la reducción de la desigualdad en salud fue moderado. ​

Marmot consideró que el *Black Report* tuvo mucha influencia en las décadas de 1980 y 1990 y permitió saber mucho más sobre las desigualdades en la mortalidad, transformándose en un estímulo para numerosas investigaciones^27^. Y si bien el *Black Report* no tuvo un impacto lineal en los años posteriores a su publicación, el trabajo de Margaret Whitehead *The health divide*^94^, publicado en 1987, y el estudio de Whitehall II del mismo año^95^ son productos del proceso iniciado por el *Black Report*^6^. Con posterioridad, se realizaron en el Reino Unido el informe *Acheson* en 1998^96^, y el *Marmot Review* en 2010^97^. 

### Ingreso mínimo para una vida sana

Después de cumplir 90 años, Morris recibió una subvención del Department of Health para investigar el ingreso mínimo para una vida sana o *minimum income for healthy living* (MIHL)^8^. Para realizar el trabajo, utilizó encuestas disponibles^98^^,^^99^^)^ y los resultados fueron publicados en una serie de artículos y comentarios^100^^,^^101^^,^^102^^,^^103^. Morris consideraba que estaban:

…en condiciones de formular y ofrecer un concepto objetivo y epistemológicamente fundamentado de las necesidades de salud, que recoge las evidencias disponibles de la investigación biomédica y social. La premisa básica del MIHL es que la investigación moderna nos está proporcionando conocimientos sólidos sobre las necesidades básicas para una vida sana personal, en nutrición, actividad física, vivienda, relaciones psicosociales e inclusión social.^103^

En los fundamentos de su planteo Morris cita el Artículo 25 de la Declaración Universal de Derechos Humanos de la ONU que señala:

Toda persona tiene derecho a un nivel de vida adecuado que le asegure, así como a su familia, la salud y el bienestar y, en especial, alimentación, vestimenta, vivienda, asistencia médica y servicios sociales necesarios; tiene asimismo derecho a seguros en caso de desempleo, enfermedad, invalidez, viudez, vejez y otros casos de pérdida de sus medios de subsistencia por circunstancias independientes a su voluntad.^104^

Para Morris la salud pública, en tanto medicina social, debe participar en el MIHL a través de la aplicación del conocimiento existente, para así mejorar las condiciones de vida y de trabajo y colaborar en crear políticas de protección social^103^. Su trabajo sobre el MIHL siguió una rigurosa metodología apoyada en evidencias científicas, y cuando examinó las necesidades de las personas mayores encontró que eran necesidades económicas que excedían los montos que prevalecían en las pensiones y beneficios estatales^7^. Para Morris, el MIHL abarcaba no solo el suficiente dinero para una alimentación saludable, sino también para poder participar en la sociedad con dignidad, lo que incluía leer un periódico y comprar regalos para los nietos. 

A pesar de la persistencia y actualidad del problema a través de los años, es indudable que la ciencia hegemónica no se siente muy cómoda con temas construidos con datos científicos tan evidentes y entendibles por la población, prefiere evidencias más sofisticadas y menos comprensibles por el conjunto social.

## A MODO DE CIERRE: ENTRE APORTES Y OLVIDOS

Para concluir, nos proponemos realizar una síntesis conceptual del pensamiento de Morris a través de las preguntas y conceptos formulados en *Health*^37^*,* y en *Uses of epidemiology*^2^, que expresan, a nuestro entender, su praxis epidemiológica que lo diferencia de las propuestas instrumentales que se encuentran en los libros más referenciados dentro de la llamada epidemiología moderna^77^^,^^79^^,^^80^. Esa síntesis conceptual de Morris la ubicamos en los siguientes tres puntos:

1. Las preguntas que formula en *Health* sobre el sentido social de la salud^37^.
2. Las características que él entiende que debería tener el diagnóstico de salud de una comunidad y las pregunta en las cuales debería basarse^2^.
3. Los siete usos de la epidemiología^2^.

La palabra “usos”, que Morris utiliza en el título de su libro más conocido, ubica a la epidemiología en una dimensión social que la transforma en una ciencia situada, basada en preguntas^73^, y enfocada en poblaciones, y no solo en casos personales o individuos^2^. Nancy Krieger recupera las concepciones de Morris y critica la epidemiología moderna, a la cual califica como no contextualizada y centrada en lo instrumental^73^, además de señalar críticamente la ausencia de reflexiones sobre la teoría epidemiológica*,* haciendo una salvedad sobre la producción de la epidemiología social latinoamericana a partir de la década de 1980^73^. 

Morris consideró la epidemiología como una ciencia histórica destinada a la resolución de preguntas prácticas, y en ese accionar demostró una actitud abierta ante otros saberes, convocando investigadores que desde diferentes disciplinas le permitían aproximarse a la complejidad de los problemas sociales. La heterogeneidad de saberes que convocó en sus proyectos indica una búsqueda transdisciplinar tan necesaria como infrecuente, y más declamada que realizada en el mundo académico. Morris fue un claro ejemplo de un pensamiento crítico ligado a la acción frente a problemas complejos. Centrado en una de las preguntas centrales de su pensamiento ya citada anteriormente: “¿cuáles son los cambios sociales que subyacen a los cambios biológicos expresados en los patrones observados”^2^. Su objetivo siempre estuvo en “cambiar a las personas en una sociedad cambiante”^2^.

La tecnificación de la medicina y de la epidemiología moderna^77^^,^^78^ anularon las capacidades de formular preguntas y de pensar en términos de problemas, lo cual se constata en las dificultades que enfrentan los egresados universitarios en formular preguntas de investigación, a pesar de la muy rica experiencia laboral que tienen, desde la cual debieran poder trabajar las preguntas que Morris encomendó responder a la epidemiología^2^.

Morris no reducía la investigación al laboratorio, no era un investigador de la ciencia experimental que se limitara a los problemas secundarios; por el contrario, buscaba las últimas instancias, de allí su preocupación por llevar, por ejemplo, la epidemiología a los servicios y sistemas de salud. 

Nancy Krieger señaló el sentido contrario al pensamiento dominante de la epidemiología y las ciencias médicas que tuvo el pensamiento de Morris:

Para Morris y sus colegas de ideas afines, el desafío era pensar en grande y en pequeño al mismo tiempo: ver los detalles de los mecanismos de las enfermedades sin perder de vista la producción social de la distribución general de las enfermedades. Fue una postura, sin embargo, que quedó cada vez más fuera de sintonía con el ascenso del individualismo biomédico y la modernidad después de la Segunda Guerra Mundial.^73^

José Ricardo Ayres, en su lectura histórica de las ideas de Morris, sostiene:

Es evidente que el fuerte movimiento de los trabajadores ingleses y el movimiento de la medicina social, que congregó nombres como Major Greenwood, Percy Stocks, Austin Bradford Hill, Richard Titmuss y Jerry Morris, además de Ryle, no fueron irrelevantes sino que desempeñaron un importante papel, tanto en términos prácticos, como la conquista de un sistema ejemplar de extensión universal de la asistencia médica, así como en el plano teórico con la producción de estudios que destacaran grandes diferencias en la distribución de la salud y de la enfermedad en términos sociales. Pero ni por esto la integración médico-sanitaria, en el sentido más ético-filosófico de Ryle, o más tecnoasistencial de Terris, llegó a experimentar mayor progreso en Inglaterra que en Estados Unidos. En los dos sentidos arriba señalados, el rescate sustancial de lo social de la salud y su correlativa interferencia estatal sobre las relaciones sociales y económicas pasaron a tener progresivas dificultades para validarse.^105^

La figura de Jeremiah Morris, como epidemiólogo, parece haber comenzado a transitar el camino del olvido. En los tiempos actuales, su figura es poco conocida por las nuevas generaciones de trabajadores de los campos de la salud, incluso de la epidemiología. Y sus preguntas, reflexiones y logros rara vez aparecen en los textos actuales de epidemiología. Ante esa situación, nos preguntamos ¿cuáles son los motivos de ese olvido o desconocimiento?, ¿su pensamiento fue superado por los desarrollos tecnocientíficos? ¿O su concepción de la epidemiología resulta incómoda para la ciencia normalizada? 

Los intereses que subyacen detrás de las dificultades señaladas por Krieger y Ayres son cada vez más evidentes, y exigen respuestas políticas que muchas veces están por fuera del campo de la salud. Pero no podemos ni debemos esperar esos cambios para actuar, los trabajadores y las trabajadoras de las áreas de salud en su conjunto pueden y deben señalar las evidentes incongruencias de muchas de las acciones de los sistemas de salud públicos, privados y de la seguridad social, tarea no menor para construir sociedades más justas, y sin desigualdades. 
